# Structural Monitoring of Glass Fiber/Epoxy Laminates by Means of Carbon Nanotubes and Carbon Black Self-Monitoring Plies

**DOI:** 10.3390/nano11061543

**Published:** 2021-06-11

**Authors:** Lorenzo Paleari, Mario Bragaglia, Francesco Fabbrocino, Francesca Nanni

**Affiliations:** 1 Department of Enterprise Engineering “Mario Lucertini”, University of Rome “Tor Vergata” and INSTM RU Roma—Tor Vergata, via del Politecnico 1, 00133 Rome, Italy; Lorenzo.paleari@uniroma2.it (L.P.); fnanni@ing.uniroma2.it (F.N.); 2 Department of Engineering, Pegaso Telematic University, 80143 Naples, Italy; francesco.fabbrocino@unipegaso.it

**Keywords:** self-monitoring, polymer matrix composite, glass-fiber-reinforced laminates

## Abstract

The health monitoring of structures is of great interest in order to check components’ structural life and monitor damages during operation. Self-monitoring materials can provide both the structural and monitoring functionality in one component and exploit their piezoresistive behavior, namely, the variation of electrical resistivity with an applied mechanical strain. In this work, self-monitoring plies were developed to be inserted into glass-fiber reinforced epoxy-based laminates in order to achieve structural monitoring. Nanocomposite epoxy-based resins were developed employing different contents of high surface area carbon black (CB, 6 wt%) and multiwall carbon nanotubes (MWCNT, 0.75 and 1 wt%), and rheologically and thermomechanically characterized. Self-monitoring plies were manufactured by impregnating glass woven fabrics with the resins, and were laminated with non-sensing plies via a vacuum-bag process to produce sensored laminates. The self-monitoring performance of the laminates was assessed during monotonic and cyclic three-point bending tests, as well as ball drop impact tests. A higher sensitivity was found for the CB-based systems (Gauge Factor 6.1), while MWCNTs (0.55 and 1.04) ensure electrical percolation at lower filler contents, as expected. The systems also showed the capability of being used to predict residual life and damage occurred under impact.

## 1. Introduction

The structural health monitoring (SHM) of structures and components is an important issue in view of both increasing users’ safety and decreasing maintenance costs. By means of SHM, in fact, it is possible to identify the structural state of a component in terms of occurring strain or stress and, in some cases, to evaluate the damage suffered during service life or exceptional loading conditions. Such information can be used to predict the component’s residual life as well as to carry out targeted maintenance interventions, thus reducing their costs. Many traditional techniques have been used periodically to perform SHM, such as acoustic emission, thermography, strain gauge, and ultrasonics, to cite a few, all of which make use of sensors placed on an external surface of the component to be monitored. Starting from the 1980s, self-monitoring materials have been proposed [1,2] which are bi-functional materials showing both structural and sensing properties. Self-monitoring materials are usually polymer composite materials where the sensing task is realized by correlating the strain or damage occurring to the structure with the variation of electrical resistance in an electrically conductive part. More commonly, electrical conductivity in polymeric composite systems is related to the presence of a carbonaceous phase, which can be in the form of long or short fibers [3,4,5], as much as in the form of nanoparticles [6,7,8,9], carbon nanotubes (CNT) or graphene nanoplatelets (GNP) [10,11,12]. In the latter cases, conductivity is achieved by the formation of a percolative network of the conductive elements [13]. Electrical conductivity is highly dependent on the nature, size and geometry of the fillers. Moreover, carbon nanotubes with different chiralities and structures, e.g., zig-zag or armchair configurations [14], as well as with different lengths and numbers of walls [15] present significantly different energy states and thus electrical and magnetic properties, ranging from semi-metallic to semi-conductive behavior [16].

Strain-sensing performance, usually referred to as piezoresistivity, is linked to the electrical resistance variation caused by an increasing strain (or damage) as a consequence of particles’ progressive physical separation and the alteration of the percolating network [17]. Based on this working principle, various self-monitoring materials have been proposed and realized through the years, such as bulk thermoplastic-based composites [9,18,19], films [20,21,22,23,24], coatings [25,26], or elastomeric or flexible sensors [27,28,29]. Much literature is present on conductive and piezoresistive systems based on thermosetting resins due to the ease of the dispersion of the nanofillers into a liquid monomer. In fact, the percolating behavior of a system is highly dependent on its polymer and filler type, synthesis method, and treatment and dimension, but also on the filler dispersion and processing method [30,31]. Just in recent years, Esmaeili et al. [10] investigated the piezoresistivity of epoxy/CNT precracked bulk samples to assess their strain and crack sensing capabilities, reporting that the systems were capable of self-monitoring crack initiation and extension inside the specimen, manifested as abrupt increases in electrical resistance. Patterned MWCNT/epoxy strain sensors are reported in [32], with the purpose of improving the difference in sensor sensitivities (i.e., gauge factors) between the tensile and compressive directions. The patterned sensors showed relatively higher sensitivities than commercial strain gauges and a narrower disparity between the gauge factors in tensile and compressive strains. Carbon black/epoxy solvent-based sensitive coatings on polypropylene substrates are reported in [33], and the suitability of the systems as small-strain sensors is investigated. Finally, Butt et al. [34] investigated the use of commercial masterbatches of single wall carbon nanotubes, MWCNT, graphene, reduced graphene oxide and nitrogen-doped graphene in several filler contents, to produce low cost, electrically conductive, and piezoresistive nanocomposites. The authors report the suitability of CNT to manufacture bulk materials with gauge factors up to 7, while graphene-based resins proved less suitable as self-monitoring materials.

Fiber-reinforced composite laminates, in particular glass-fiber-reinforced composites, can also benefit from the structural health monitoring functionality granted by piezoresistive materials. Tzounis et al. [35] reports the deposition of CNTs directly onto the surface of glass fibers via water-based blade coating, resulting in a strongly orthotropic electrically conductive system with remarkable damage-sensing capability upon delamination. Bragaglia et al. [26] report the development of GNP, MWCNT and hybrid GNP + MWCNT epoxy resins applied as coatings onto the external surface of glass-fiber-reinforced composites. Gauge factors up to 10.3 were reported, and the sensitivities of the materials were related to the nanofillers’ interparticle distance using a theoretical model. In situ monitoring of through-thickness strain in glass-fiber-reinforced laminates was investigated in [36]. CNT/epoxy resins were used to fabricate the composites via hand lay-up, and the through-thickness piezoresistivity to transversal loads was reported. Finally, Rhegat et al. [37] developed graphene-coated glass fabrics to fabricate self-monitoring laminates, reporting high sensitivity with gauge factors up to 14.6 and reliable sensing performance during cyclic testing.

**Figure 1:**
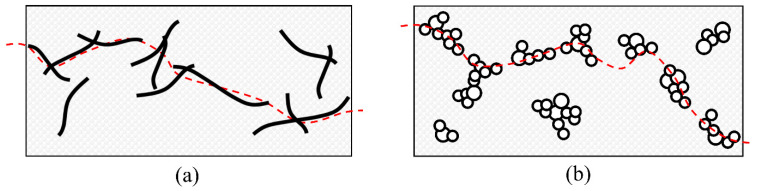
Structure of the percolating network in (**a**) CNT- and (**b**) CB-based systems on glass-fabric substrate. Red dashed line represents the conduction path across the continuous network.

The aim of this work is to develop smart self-monitoring glass woven fabrics to be used as individual strain-sensing plies in the preparation of composite laminates. Such smart fabrics were realized by pre-impregnating selected areas of the fabric with CB- or MWCNT-filled epoxy resins by means of the doctor blade technique. A schematic of the self-monitoring plies is depicted in Figure 1, where the red dashed line represents the conduction path across the percolating network, composed of ohmic conduction through contacting filler particles and tunneling conduction across the gap between particles or agglomerates. The nanocomposite resins were characterized in terms of their rheology and thermomechanical properties. The pre-impregnated sensitive plies were stacked together with neat epoxy/glass fabric plies to form self-monitoring composite laminates that were tested under monotonic and cyclic three-point bending and impact tests. Self-monitoring performances were evaluated by measuring the electrical resistance variation during the mechanical tests. Moreover, the presence of a residual electrical resistance after cyclic and/or impact loading was measured and can possibly be correlated with the residual life of the material.

## 2. Materials and Methods

### 2.1. Resins Preparation and Characterization

The conductive composite resins were manufactured using a two-part epoxy system constituted of ElanTech EC 157 (Elantas, Wesel, Germany) bisphenol-F-epichlorohydrin monomer and amine-based curing agent ElanTech W 152 (Elantas, Wesel, Germany) [38] in the weight ratio of 100:30. Two different conductive nanofillers were used alternately: (i) carbon black powder (CB, Evonik XE2B, Essen, Germany), with average particle size of 30 nm, dibutyl phthalate (DBP) absorption 411 mL/100 g, and surface area 1000 m^2^/g as declared by the producer; and (ii) multiwalled carbon nanotubes (MWCNTs NC3150, Nanocyl, Sambrevuille, Belgium), with average diameter of 9.5 nm, length <1 µm, purity <95%, and oxides <5%.

Carbon black, in the content of 6 wt%, was dispersed in the monomer by mechanical mixing with a planetary mixer Thinky ARE-250 (Thinky, Tokyo, Japan) using a mixing cycle of 20 min at 2000 rpm, followed by a 5 min defoaming cycle at 1200 rpm.

MWCNTs, in the compositions of 0.75 and 1 wt%, were first dispersed in ethanol via sonication using a Vibracell VC750 tip sonicator (Sonics, Newtown, CT, USA) for 20 min with 750 W of power. Then the monomer was added and the suspension was kept under magnetic stirring up to solvent evaporation.

For all compositions, the curing agent was added and mechanically stirred prior to curing.

Filler contents, reported in Table 1, were chosen according to previous experimentations, where it was found that in these conditions both systems are in a conductive state, i.e., above percolation [7,26,39]. The viscosity of the uncured resins was measured at room temperature (RT) by means of a Kinexus Lab+ rheometer (Malvern Panalytical, Malvern, UK), using a frequency sweep configuration (shear rate 10–100 s^−1^) at 25 °C. The thermomechanical properties of the cured resins were measured via dynamic thermal analysis (DMA) using a Triton 2000 DMA (Perkin Elmer, Walfam, MA, USA) from RT to 160 °C with a heating rate of 2 °C/min in the single-cantilever configuration.

### 2.2. Glass Fabric Laminates Preparation

**Figure 2:**
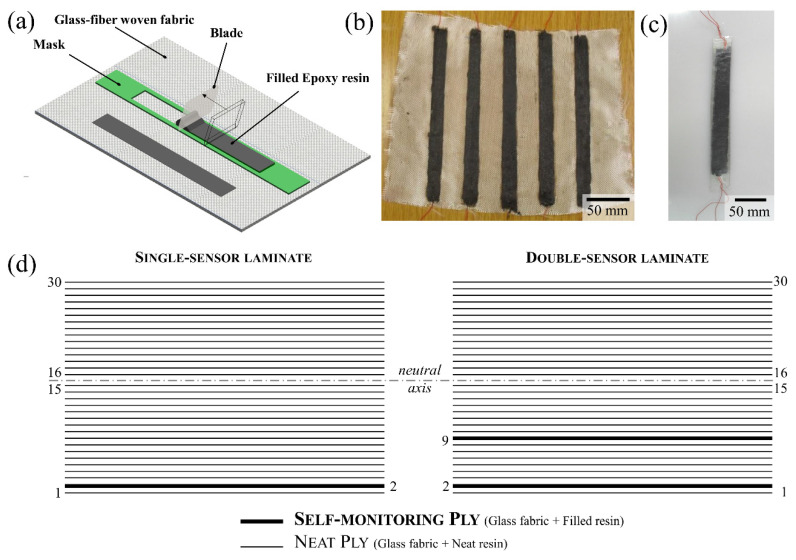
(**a**) Doctor blade technique schematics, (**b**) impregnated self-monitoring plies, (**c**) sensored laminate and (**d**) stacking sequence of laminates (dashed line represents the neutral axis).

After the addition of the curing agent, the doctor blade technique was used to deposit the filled resins on glass satin woven fabrics (type G1, 80 g/m^2^, Shaller Compositi, Mantova, Italy) in 200 × 15 mm^2^ rectangular stripes by means of a mask (Figure 2b). During the doctor blade process, the filled resin was deposited on the substrate by a moving blade, generating a thin coating [40] as sketched in Figure 2a. An electrical connection was provided at each end of the conductive strips with thin solid copper wires and conductive silver paint. The coated plies are henceforth referred to as “self-monitoring plies” (SMP).

One or two SMPs were stacked with neat glass fabric plies to form 30-ply laminates (Figure 2c) by means of the vacuum bagging technique. Figure 2d shows the stacking sequence of the single- and double-sensored laminates. The SMPs’ position in the stacking sequence was chosen in order for the external sensor to experience maximum strain during the bending loading of the laminate, while providing electrical insulation with the outermost non-conductive ply; the internal sensor, when present, was placed halfway between the external sensor and the neutral axis, thus experiencing half the maximum strain. The stacked laminates were allowed to cure for 24 h at RT and post-cured at 60 °C for 10 h. Table 2 summarizes the nomenclature and composition of produced samples.

The initial electrical resistance of the self-monitoring plies was measured using a digital multimeter (*Keithley* DMM 2700).

### 2.3. Sensored Laminates Testing

The laminates were mechanically tested via monotonic and cyclic three-point bending tests using an Instron 5569 universal machine (Instron, Norwood, MA, USA), equipped with a 50 kN load cell and a crosshead speed of 2 mm/min. Cyclic tests were carried out following triangular deflection ramps with an increasing amplitude of 2 mm per cycle. Ball drop impact tests were conducted using a custom impact facility, designed according to ASTM F736 standards, with a 550 g steel ball falling from a height of 70 cm (3.77 J of energy) and impacting on the center of the sample mounted in a three-point bending configuration. During mechanical and impact tests the electrical resistance variation was measured by means of a digital multimeter (Keithley DMM 2700, Keithley Instruments, Solon, OH, USA). Samples’ morphology was observed on gold-sputtered sections by means of a field emission scanning electron microscope (FEG-SEM, LEO Supra 35, Zeiss, Jena, Germany).

## 3. Results and Discussion

### 3.1. Resin Characterization

**Figure 3:**
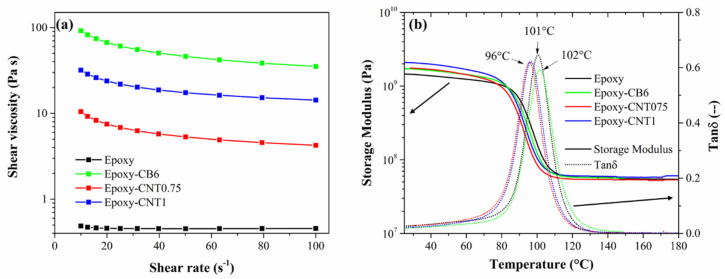
(**a**) Rheological results and (**b**) storage modulus (solid lines) and loss factor (tanδ, dotted lines) as function of temperature for resin systems.

The rheology curves of the resin/filler uncured systems are presented in Figure 3a. The neat Epoxy resin shows low viscosity values and mostly Newtonian behavior, presenting a constant viscosity with an increasing applied shear rate. On the other hand, the addition of both nanofillers led to an increase in viscosity and the modification of the rheological behavior. The composite resins, indeed, show a shear thinning behavior, as the viscosity decreases with an increasing shear rate. An increase in shear rate is known to cause the rupture of aggregates and redistribution of the filler particles, thus decreasing the resin viscosity. The Epoxy-6CB viscosity results were much higher than the other systems throughout the shear rate range, which is easily explained considering the high filler content needed to reach electrical percolation. In fact, the rheological behavior of a nanocomposite resin is highly dependent on the nature, size, dispersion and content of the filler [41,42].

Figure 3b shows the DMA results of the cured resin systems, in terms of storage modulus (E’) and tanδ as functions of temperature. Comparing the composite resins to the neat Epoxy, a small increase in storage modulus was detected throughout the temperature range. This effect is ascribed to a decrease in polymer chain mobility due to the interfacial interaction between the nanoparticles and the matrix, which causes an increase in stiffness both in monotonic and dynamic conditions. The increase in modulus is, however, limited, due to the low filler contents used, with values at RT ranging from 1.45 to 2.08 GPa for Epoxy and Epoxy-CNT1, respectively. The glass transition temperature (Tg) of the neat Epoxy, measured as the tanδ peak, was 101 °C, which is in good accordance with the data sheet values provided by the producer. Epoxy-CB6 presented a slight increase in Tg, which is again ascribable to a restriction in segmental chain mobility due to the presence of the filler nanoparticles. Epoxy-0.75CNT and Epoxy-1CNT, on the other hand, presented almost identical transition temperatures which are slightly lower than the neat Epoxy value. This behavior is in accordance to literature [43] and may be related to the higher thermal conductivity of the CNT-filled composites, which leads to a faster transition during the dynamic heating scan and prevails over the stiffening effect. Furthermore, the latter effect also results in a lower energy dissipation and overall lower damping properties for all filled resins, as shown by the decrease in the peak tanδ absolute values when compared to the neat Epoxy. The E’ values at 25 °C and glass transition temperatures resulting from DMA tests are reported in Table 3.

### 3.2. Electrical and Morphological Characterization of Self-Monitoring Plies

The electrical resistance values for the produced SMPs are presented Table 3. The conductive plies made with CNT-based resins showed conductivity values similar to those based on CB although with a much lower filler content, in agreement with the literature [44]. CNT-based systems, indeed, presented percolation thresholds at a much lower filler content [45] thanks to their very high aspect ratio [46,47]. Furthermore, the low filler content allows for a less viscous resin which facilitates fiber impregnation and is thus responsible for a more reproducible fabrication process compared to CB-based samples, as testified by the lower variability found in the SMPs resistance (see standard deviations in Table 3).

**Figure 4:**
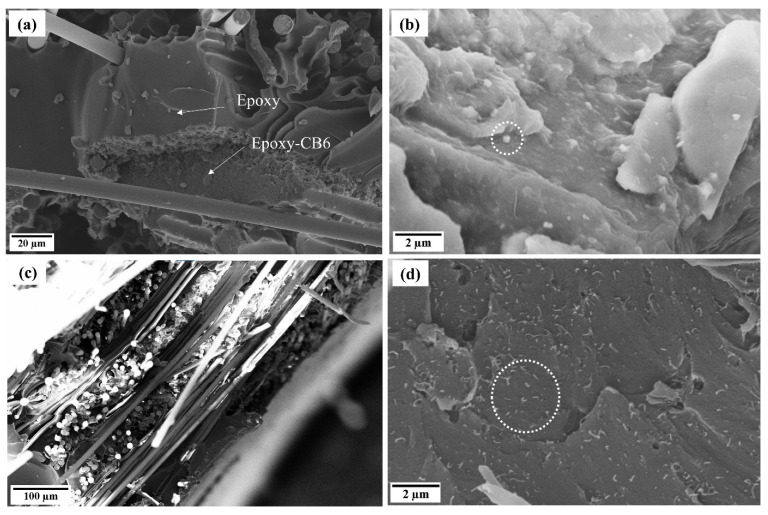
SEM micrographs of (**a**,**b**) CB6_1 and (**c**,**d**) CNT1_1 fractured laminate.

SEM observations of the fractured laminates’ morphology are shown in Figure 4. Generally, good compatibility between filled and neat resin was ensured in the laminates (Figure 4a,c). A good dispersion of the CNTs (circled with dotted line) in the matrix was achieved (Figure 4d). Carbon nanotubes appear to be randomly and homogenously distributed within the epoxy matrix. On the other hand, in the case of CB, the presence of some aggregates (round particles, circled with dotted line) in the matrix was detected (Figure 4b).

### 3.3. Self-Monitoring Performances of the Laminates

**Figure 5:**
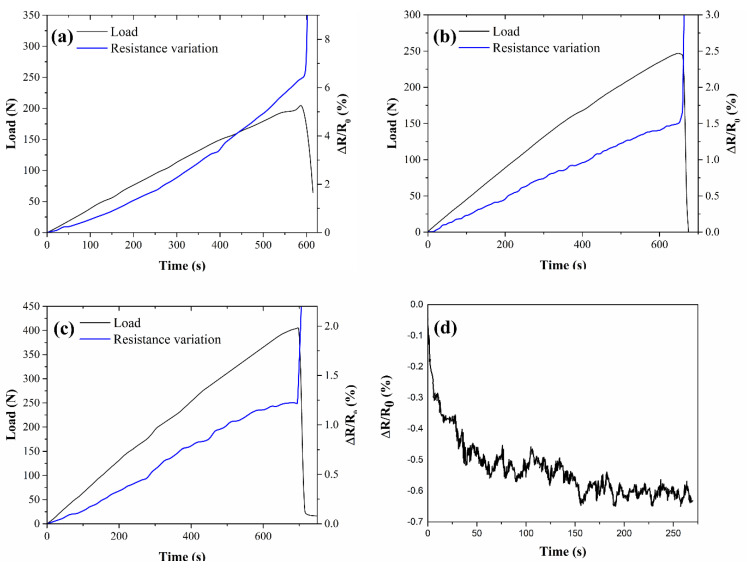
Self-monitoring behavior of (**a**) CB6_1, (**b**) CNT0.75_1, and (**c**) CNT1_1 under monotonic bending tests; and (**d**) electrical acquisition of CNT0.75_1 in the unloaded state.

Figure 5 reports the self-monitoring characteristics of the laminates with one SMP, in terms of applied mechanical load and electrical resistance variation as functions of time. The latter is evaluated as the variation in resistance relative to the unloaded state (ΔR/R_0_ = (R−R_0_)/R_0_). CB-based samples (Figure 5a) showed piezoresistive performances, with an exponential increase in the electrical resistance with time and, thus, deformation. Such behavior is typical of tunneling conductivity in carbon-black-loaded composites [48,49]. In the case of CNT samples (Figure 5b,c), the electrical resistance variation followed a much more linear trend than the case of CB samples, as the material expressed an ohmic predominant behavior. Nevertheless, the authors believe that tunneling is still the predominant mechanism of conductivity, as evidenced in the case of the CNT0.75_1 samples, where an increase in electrical conductivity was recorded even in the unloaded state, up to an asymptotic value (Figure 5d). This can be ascribed to the fact that in predominantly tunneling conductors, merely the heat generated by current flow promotes electron jumping, hence, helping conductivity [50]. In the 1% CNT sample, on the other hand, this effect was not present, since the higher filler content grants a percolating system with a predominantly ohmic conduction.

The gauge factors (GFs), which measure the sensitivity of the strain-sensing materials to the applied strain, have been calculated by applying the definition GF = (ΔR/R_0_)/ε, where (ΔR/R_0_) is the relative electrical resistance variation and ε is the mechanical strain (mm/mm), calculated from three-point bending test. In the case of the CB6_1 samples, the average GF was found to be around 6.1, while for CNT0.75_1 and CNT1_1 they were 0.55 and 1.04, respectively. The gauge factor found for the CNT-based samples was in line with the literature, in which sensitivities between 0.5 and 2 are usually found for Epoxy-CNT systems with similar filler contents [10,11,51,52,53], as well as those which are CB-based [33,54].

**Figure 6:**
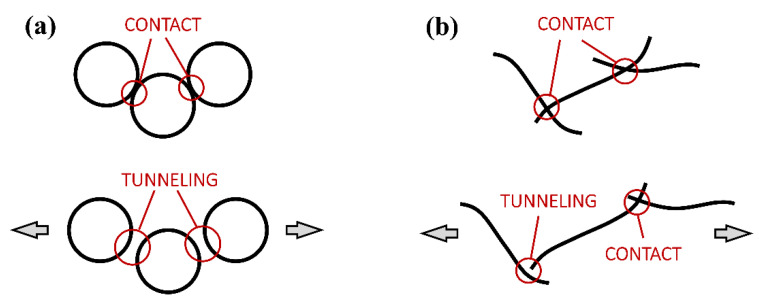
Mechanism for the piezoresistivity of (**a**) CB and (**b**) CNT systems.

The GFs can be directly related to the microscopic origin of conductivity, that, in turn, depends on the fillers’ geometrical shape (or that of the aggregates), intrinsic conductivity, initial distance among the particles or aggregate, and the area of conductivity between two overlooking or touching particles. The increase in resistance at increasing strain is due to the decrease in the number of conductive paths [55], as well as the rearrangement of the circuit formed by aggregates [48]. Both CB6_1 and CNT1_1 had more or less the same initial electrical resistance (400–480 kΩ) that, however, originates from very different internal microstructures and amounts of filler. In fact, CB is present in quite large amounts (6 wt%) that is distributed in highly branched aggregates (the used carbon black has a high surface area and oil absorption number [56]). SEM micrographs (Figure 4b) show that the average distance between the rounded aggregates is relatively high, so that a small deformation rapidly leads to an increase in electrical resistance, resulting in high GFs (Figure 6a). On the contrary, in the case of CNT, the same level of conductivity was reached with very few nanotubes (0.75–1 wt%) since their high aspect ratio and geometrical shape promote the formation of a percolative path. Nevertheless, for the same reason, statistically more deformation is required to separate overlooking or touching CNTs (Figure 6b), resulting in a smaller GF.

**Figure 7:**
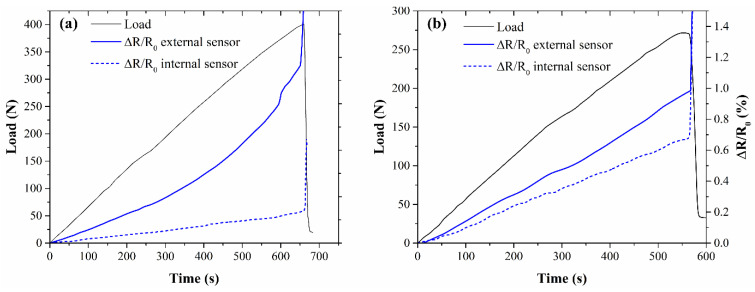
Self-monitoring behavior of (**a**) CB6_2, (**b**) CNT0.75_2 under monotonic bending tests.

As presented in Figure 7, the laminates containing two SMPs showed the self-monitoring behavior of both sensing laminae at the same time. As expected, the internal lamina, closer to neutral axis, experienced less deformation and, thus, lower electrical resistance variation. This is particularly evident in the CB-loaded samples (Figure 7a), which presented high GF, while the small GF associated with CNT systems caused a lower variation of electrical resistance between the two self-monitoring plies within the same sample (Figure 7b).

**Figure 8:**
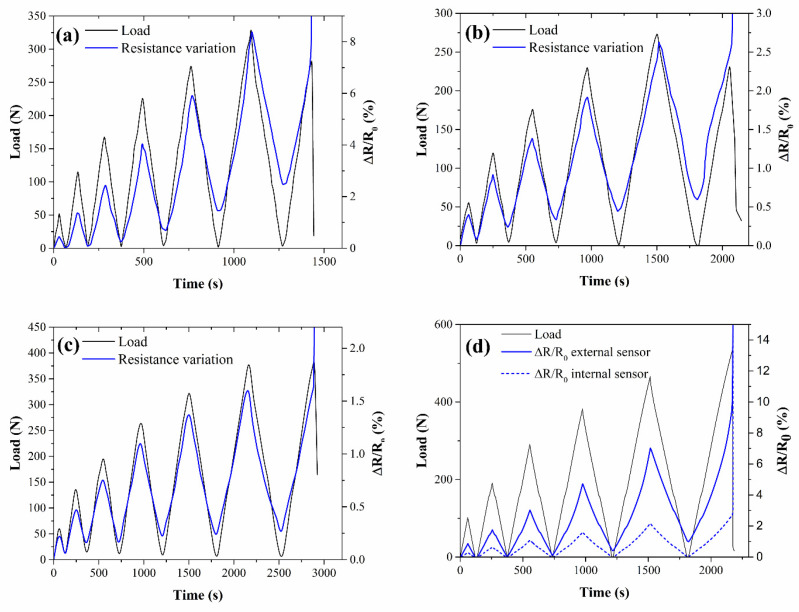
Self-monitoring behavior of (**a**) CB6_1, (**b**) CNT0.75_1, (**c**) CNT1_1, and (**d**) CNT0.75_2 under cyclic bending tests.

The results of the cyclic bending tests on the one-SMP laminates and a selected two-SMP sample are presented in Figure 8. All curves show that the electrical resistance closely follows the applied stress, and thus strain, with no recorded electrical delay, as also found in literature with similar self-monitoring systems [11,37]. After some cycles, however, a residual electrical resistance variation is present, which is ascribed to irreversible changes in the material, such as cracking and/or the plastic deformation of the resin [57]. No shouldered or double peak was detected, as opposed to some cases in the literature [11,58] in which the competition between two opposed effects, i.e., network destruction and reconstruction, led to a non-monotonic response to the loading or unloading.

Maximum load and electrical resistance variation values measured via monotonic and cyclic bending tests for all samples are reported in Table 4.

### 3.4. Self-Monitoring Performances on Impact

**Figure 9:**
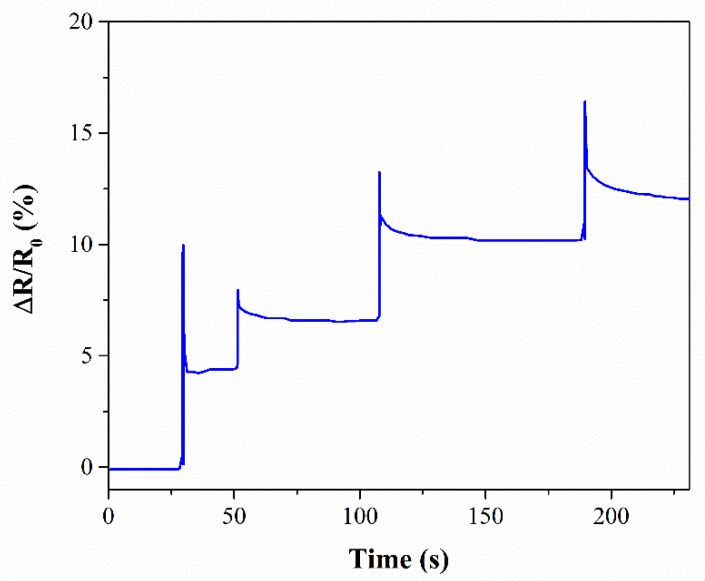
Electrical resistance variation vs. time of CB6_1 under subsequent impacts.

A representative curve (CB_1 sample) of the self-monitoring performances of the sensored laminates upon subsequent impacts up to fracture is reported in Figure 9. The samples showed instant electrical resistance variations at the moment of the impact, followed by partial recovery to a residual permanent variation, in perfect agreement with the literature on impact testing on similar systems [59,60,61]. The latter is attributed to the permanent damage of the material.

The variation of electrical resistance, indeed, can be thought of as composed of an enthalpic term (related to the elastic energy which is recovered after the impact) and an entropic term (the energy lost in form of delamination, ply damage, or irreversible changes in the microstructure of the material). It seems possible to speculate that the energy balance during the impact event could be expressed as in Equation (1):

E_impact_ = E_rel_ + E_abs_ + E_f_ (1)

where E_impact_ is the total amount of energy of the ball at impact, E_rel_ is the enthalpic energy released after impact, E_abs_ is the enthropic energy absorbed as general damage, and E_f_ the entropic energy dissipated by friction during the ball fall. As a first hypothesis, E_f_ can be considered negligible, being much less than the other terms.

Considering that E_impact_ is known during the test and that E_rel_ does not concur with the damage, it could be possible to calibrate the system to establish a correlation between measured residual electrical resistance and the damage caused to the laminate, with the latter evaluated by means of image analysis techniques. To this end, a new experimentation has to be carried out. This possibility is particularly significant for common applications of composite laminates, which are particularly sensitive to impact damage, with a view to quantify and, hence, predict residual life. In particular, the use of a grid of sensitive plies in a laminate may be useful not only to detect and quantify damage, but also to localize it and evaluate the number of permanently affected plies, as it is known that several failure mechanisms concur with impact damage propagation in laminates, such as fiber breakage, matrix cracking, fiber debonding, fiber pull out and delamination [59].

## 4. Conclusions

Self-monitoring composite materials were manufactured and tested by preparing sensitive plies via the doctor blade technique with epoxy resin filled with either carbon black or MWCNT. The resin systems showed shear thinning behavior and thermomechanical properties which closely reflect the nanocomposites’ morphology. The sensored laminates were tested either under monotonic and cyclic three-point bending tests. Both systems showed self-monitoring abilities, but with different sensitivities. In particular, CB laminates had a superior gauge factor to that of the MWCNT samples and this was explained by considering the higher aspect ratio of the CNTs that, while insuring percolation at a lower filler level, allows nanotube separation at a higher strain. The system showed to be potentially successful at predicting residual life under impact.

## Figures and Tables

**Table 1:** Resin systems compositions.

Resin Name	Filler Type	Filler Content (wt %)
Epoxy	none	-
Epoxy-CB6	CB	6
Epoxy-CNT0.75	MWCNT	0.75
Epoxy-CNT1	MWCNT	1

**Table 2:** Nomenclature of laminate samples.

Self-Monitoring Plies		Laminate Sample Name
Resin Type	Number of SMPs	
Epoxy-CB6	1	CB6_1
	2	CB6_2
Epoxy-CNT0.75	1	CNT0.75_1
	2	CNT0.75_2
Epoxy-CNT1	1	CNT1_1

**Table 3:** DMA results of bulk cured resin and electrical resistance of impregnated SMP for each resin system.

	Dynamic Mechanical Analysis		Average SMP Electrical Resistance (R_0,_ kΩ)
Resin System	E’ at 25 °C (GPa)	Tg (°C)	
Epoxy	1.45 ± 0.06	100.6 ± 0.3	-
Epoxy-CB6	1.72 ± 0.07	101.7 ± 0.5	402 ± 38
Epoxy-CNT0.75	1.76 ± 0.10	96.0 ± 1.0	611 ± 8
Epoxy-CNT1	2.08 ± 0.09	96.6 ± 0.8	4805

**Table 4:** Mechanical and self-monitoring properties of CB and CNT systems.

Sample	Monotonic Bending		Cyclic Bending	
	Max Load (*n*)	Max ΔR/R_0_ (%)	Max Load (*n*)	Max ΔR/R_0_ (%)
CB6_1	204.5	6.49	328.5	8.38
CNT0.75_1	246.7	1.69	273.1	2.64
CNT1_1	404.7	1.25	399.1	1.68

## Data Availability

The data presented in this study is available on request from the corresponding author.
